# On the Role of Core Exercises in Alleviating Muscular Fatigue Induced by Prolonged Sitting: A Scoping Review

**DOI:** 10.1186/s40798-025-00816-x

**Published:** 2025-02-21

**Authors:** Banafsheh Amiri, David G. Behm, Erika Zemková

**Affiliations:** 1 Department of Biological and Medical Sciences, Faculty of Physical Education and Sport, Comenius University in Bratislava, Bratislava, Slovakia; 2 School of Human Kinetics and Recreation, Memorial University of Newfoundland, St. John’s, NL Canada

**Keywords:** Electromyography, Muscle activity, Sedentary, Tensiomyography, Workplace

## Abstract

**Background:**

Prolonged sitting induces fatigue in deep trunk muscles, thereby decreasing spinal support and increasing stress on the spine. Core exercises effectively facilitate recovery from trunk muscle fatigue based on evidence from subjective outcomes; however, there is a lack of systematic investigation into localized muscle activity specifically related to prolonged sitting. Therefore, this scoping review summarizes the evidence on the effects of core exercises in alleviating trunk muscular fatigue induced by prolonged sitting, focusing on objective outcomes such as electromyography (EMG) and tensiomyography.

**Methods:**

Articles published between January 2010 and February 2024 were sought in PubMed, Scopus, Web of Science, MEDLINE, and Cochrane Library, as well as Elsevier, SpringerLink, and Google Scholar. The findings were reported following the guidelines provided by the PRISMA-ScR checklist.

**Results:**

Out of 106 papers initially identified, eight met the inclusion criteria. During core exercises, fatigued trunk muscles exhibit an increase in EMG root mean square (RMS) values. In the post-intervention (from 9 days to 8 weeks), tensiomyography outcomes of the erector spinae muscles significantly improved, whilst EMG results were insignificant. The contraction times of both the left and right erector spinae balanced out, indicating a harmonizing effect of the exercise. Regarding the recovery of other trunk muscles, only the transversus abdominis and internal oblique muscles exhibited a significant increase in the EMG median frequency that decreased over prolonged sitting.

**Conclusions:**

Core exercises are effective in alleviating muscular fatigue caused by prolonged sitting. This can be observed from significant changes in EMG and tensiomyography parameters during exercise and after the training protocol. Intervention studies have primarily targeted the recovery of the erector spinae muscles, with less attention on other trunk muscles. Although significant improvements in tensiomyography results for the erector spinae can be observed after core exercise intervention, the impact on EMG remains uncertain.

**Key Points:**

1. Core exercises have an acute effect on reducing muscle fatigue caused by prolonged sitting.
2. Core exercise intervention studies, primarily targeting the erector spinae muscles, significantly improve tensiomyography outcomes, but evidence for electromyography outcomes is lacking. In addition, few studies have analyzed the electromyography mean frequency and root mean square values, which are crucial in assessing muscle fatigue and recovery.
3. Only a few studies have investigated the effectiveness of core exercises in restoring other trunk muscles, such as the transversus abdominis and multifidus, following prolonged sitting fatigue.

## Background

Sedentary behavior refers to activities that are performed at or just above the resting metabolic rate (1–1.5 METS) [1]. In 28 European countries, an estimated one-third of adults under the age of 65 are characterized as inactive [2]. A major factor in adults' sedentary behavior is their daily employment [3]. Technological advancement has had a global impact on occupational sedentariness, which has increased over time [4, 5]. The growing reliance on computers in the workplace has led to extended periods of sitting for employees, with fewer breaks [6]. On average, office workers sit for 6.6–10.0 h per day [3]. Because of the lower center of gravity and a wider base, sitting is a comfortable posture that consumes little energy [7]. The prevalence of this condition increased during the COVID-19 pandemic, with remote work from home leading to prolonged sitting [8, 9].

During prolonged sitting, continuous contraction of the core muscles can lead to deep trunk muscle fatigue [10]. Under fatigue, increased activity in superficial trunk muscle reduces support to the spine and increases stress on ligaments and intervertebral discs [10]. The reduction of disc height increases the amount of compression on sensitive spinal structures. This may stimulate nociceptor activity, which could contribute to the development of pain [10]. Research indicates that 34 to 51% of office workers experienced low back pain in the past year, with approximately 20 to 23% reporting new episodes during a one-year monitoring period [12]. The COVID-19 pandemic has significantly increased the occurrence of lower back pain as the most prevalent population problem [13]. Low back pain has been identified as the leading cause of disability worldwide, measured in years lived with disability, and ranks sixth in terms of overall burden, based on disability-adjusted life years [11].

Due to the strong correlation between low back pain and weak or imbalanced trunk muscle corsets [14], researchers investigated various interventions aimed at reducing trunk muscle fatigue [15]. In particular, exercise programs conducted in workplace settings are beneficial in this regard [16–18]. Among them, core exercises have achieved substantial popularity in recent years [10, 16, 17, 19–24]. The purpose of these exercises is to restore normal muscle function to increase spinal stability, improve neuromuscular control within the lumbopelvic region, increase intersegmental stiffness, and prevent injury to the lumbar spine [11]. These exercises play a crucial role in reducing muscle fatigue induced by prolonged sitting [10, 16, 19–24].

Muscle fatigue from prolonged sitting is commonly assessed using surface electromyography (EMG) [25–28]. Various indexes have been proposed to analyze EMG signals and quantify the degree of muscle fatigue, such as median frequency (MDF), root mean square (RMS), fractal dimension, and average rectified value [29–32]. These indexes aim to capture different aspects of muscle fatigue related to physiological changes within the muscle [15]. Fractal dimension, for instance, has been found to be highly sensitive to detecting the synchronization of recruited motor units, reflecting central fatigue [33]. On the other hand, frequency domain indexes like MDF or mean frequency (MNF) are associated with the nerve conduction velocity of motor unit action potentials, mainly reflecting peripheral fatigue [30, 34]. Evaluating the effects of various physical exercises on reducing muscle fatigue using different EMG indexes can provide insights into the potential benefits of each intervention and shed light on the underlying physiological mechanisms [15].

Tensiomyography is another widely-used noninvasive method for assessing muscle fatigue in a static posture [22]. It assesses muscle state changes by examining the displacement and contraction time of muscle fibers induced by a 1 ms electrical stimulation (ranging from 0 to 100 mA) of a single muscle fiber bundle [22]. The advantage of tensiomyography is its lower sensitivity to external noise and also the fact that it is not affected by skin resistance or sweating [22]. It is considered a valid technique for evaluating muscle fiber composition [22].

In recent systematic reviews, researchers investigated the effect of exercise interventions on reducing neuromuscular fatigue [15, 35, 36]. However, two review studies primarily relied on subjective measures, such as perceived fatigue and soreness [35, 36], which may not accurately reflect localized muscle fatigue. Only one review study employed objective measures, such as EMG [15]. Additionally, all three systematic reviews primarily focused on post-exercise fatigue and did not consider fatigue resulting from prolonged sitting [15, 35, 36]. Furthermore, while several studies discussed interventions such as active recovery, compression, massage, and stretching, a significant gap remains in research regarding the effectiveness of core exercises for alleviating fatigue [37–41].

## Objectives

This scoping review aims (i) to summarize the evidence on the effects of core exercises in alleviating trunk muscular fatigue induced by prolonged sitting, focusing on objective outcomes such as EMG and tensiomyography and (ii) to identify gaps in the existing literature and suggest recommendations for future studies on this topic.

## Methods

### Protocol and Registration

This study was drafted using the PRISMA-ScR (Preferred Reporting Items for Systematic Reviews and Meta-Analyses Extension for Scoping Reviews) checklist [42]. The process was based on the ‘Population–Concept–Context (PCC)’ framework recommended by the Joanna Briggs Institute for Scoping Reviews [43]. Moreover, the protocol was preregistered on 3 April 2024 using the Open Science Framework (OSF) Registries (https://doi.org/10.17605/OSF.IO/7VFZ6).

### Eligibility Criteria

The inclusion criteria comprised peer-reviewed English-language articles published from January 2010 to February 2024. Grey literature was excluded, including books, theses, case reports, abstracts, and conference papers.

According to the PCC framework, as delineated in Table 1, the population encompassed individuals who were sedentary or experienced prolonged periods of sitting, without restrictions based on age, sex, or specific health conditions (e.g., low back pain sedentary, or healthy sedentary). Prolonged sitting was typically defined as sitting for at least two hours on any working day [25]. The concept involves the studies assessing the impact of core exercises on trunk muscle fatigue induced by prolonged sitting through objective methods. Studies from diverse workstation settings, such as community, workplace, residential, and hospital settings, were eligible for inclusion. The context is international, with no restrictions on location, time frame, or environment.

**Table 1:** ‘Population–Concept–Context (PCC)’ framework

PCC element	Description
Population	Individuals who were sedentary or experienced prolonged periods of sitting, without restrictions based on age, sex, or specific health conditions (e.g., low back pain sedentary, or healthy sedentary). Prolonged sitting was typically defined as sitting for at least two hours on any working day [25]
Concept	The studies assessed the impact of core exercises on trunk muscle fatigue induced by prolonged sitting through objective methods
Context	Studies from diverse workstation settings, such as community, workplace, residential, and hospital settings, were eligible for inclusion. International, with no restrictions on location, time frame, or environment

### Information Sources

The search was conducted with PubMed, Scopus, Web of Science, MEDLINE, and Cochrane Library, as well as Elsevier, SpringerLink, and Google Scholar. Moreover, manual searches were conducted on the references cited within the reviews to identify any additional pertinent studies.

### Search Strategy

The search strategy included combinations of the following terms related to sedentary behavior, core exercises, evaluation of muscle fatigue and activity, and core muscles:

1. Sedentary behavior:

"Sedentary behavior" AND "prolonged sitting" AND "workplace" AND "office worker" AND "employees" AND "workstation";

2. Core exercises:

"Core stabilization exercise" AND "abdominal drawing-in maneuver technique" AND "lumbar stabilization exercises" AND "Pilates" AND "yoga" AND "stability ball exercises" AND "medicine ball exercises" AND "TRX suspension training" AND "diaphragmatic breathing";

3. Evaluation of muscle activity:

"Electromyography OR 'EMG'" AND "muscle activation pattern" AND "tensiomyography OR 'TMG " AND "muscle activity" AND "muscle fatigue" AND "neuromuscular activity";

4. Core muscles:

“Core muscle” AND "rectus abdominis" AND "erector spinae" AND "transverse abdominis" AND "internal oblique" AND "external oblique" AND "latissimus dorsi" AND "iliocostalis lumborum pars thoracis" AND "multifidus".

## Results

### Selection of Sources of Evidence

**Fig. 1:**
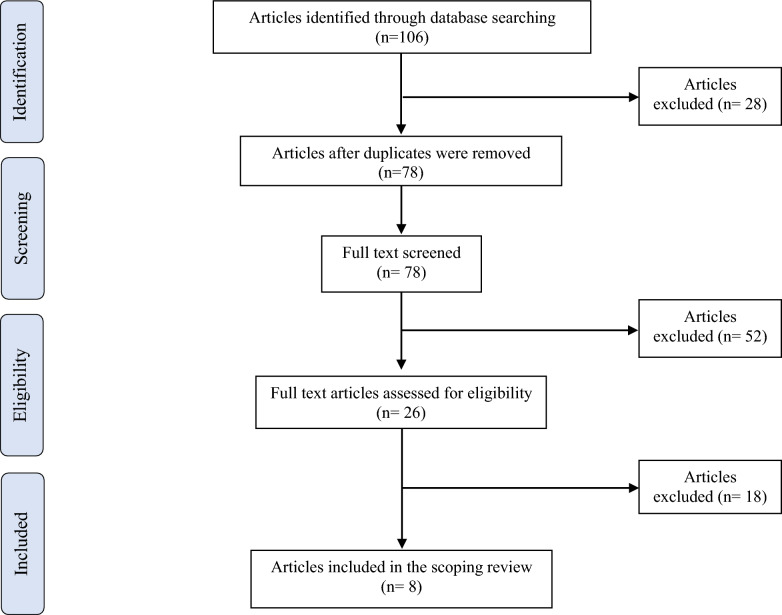
PRISMA diagram

A total of 106 articles were identified during the initial database search. Twenty-eight (28) duplicates were removed resulting in 78 sources to be screened. After a preliminary review and evaluation for eligibility, 52 studies that did not meet the inclusion criteria were excluded. Among the 26 remaining articles, 8 were included in this scoping review (Fig. 1: PRISMA diagram).

### Data Items and Charting Process

Data extraction for the chosen articles was carried out using a standard Excel spreadsheet. One reviewer, BA, was in charge of data extraction, while another reviewer, EZ, conducted a thorough review to ensure the accuracy of the extracted data. The data extracted encompassed publication information (such as study location, study design, and publication year), the primary health outcome under investigation, participant demographics (including sample size, age, sex, sport, and spine-related issues), the research methodology employed, and the key findings related to health outcomes.

### Temporal Precursor Variables

Tables 2, 3, 4 present summaries and frequency of the basic characteristics of the selected articles. The participants in all studies were young adults aged 20–40 years.

**Table 2:** Frequency of studies regarding sex, health status, and muscles investigated

Studies investigating acute effects of core exercise on muscle activity									
Sex	Males			Females				Both sexes	
# of studies	–			–				3	
References	–			–				[16, 19, 20]	
Health Status	Sedentary	Sedentary postures				Healthy adults			Low back pain
# of studies	2	1				1			2
References	[16, 19]	[20]				[20]			[16, 19]
Muscles examined	Erector Spinae	Internal Obliques	Rectus Abdominus		Multifidus	External Obliques	Transversus Abdominus		Iliocostalis Lumborum
# of studies	1	3	3		3	1	2		2
References	[20]	[16, 19, 20]	[16, 19, 20]		[16, 19, 20]	[20]	[16, 19]		[16, 19]

Studies investigating effects of core exercise interventions on muscle activity									
Sex	Males			Females				Both sexes	
# of studies	1			2				2	
References	[22]			[21, 23]				[10, 24]	
Health Status	Sedentary	Sedentary postures				Healthy adults			Low back pain
# of studies	4	1				3			2
References	[10, 21–23]	[24]				[22–24]			[10, 21]
Muscles examined	Erector spinae	Internal obliques	Rectus abdominus		Multifidus	External obliques	Transversus abdominus		Iliocostalis lumborum
# of studies	4	2	2		1	1	1		1
References	[21–24]	[10, 24]	[10, 21]		[10]	[24]	[24]		[10]

^#^The number of studies conducted in each category

**Table 3:** Summary of studies investigating acute effects of core exercises on muscle activity

Participants	Intervention	Data collection	Equipment & variables	Measured muscles	Muscle activity response to core exercises
Study: Saiklang et al. [19]					
Sample Size: 24 participants (12 males and 12 females); Age Range: 20 to 35 years old; Inclusion Criteria: CLBP patients; Low levels of activity limitation; Reported sitting for at least 2 h on any working day	-Experimental Group: *Core Exercise Types:* abdominal drawing-in maneuver technique; *Duration:* performed the technique for 1 min and repeated it three times (at 12–13, 25–26, and 38–39 min) during a 41-min prolonged sitting time Control Condition: Sitting without intervention	Timepoint: during protocol Method: the values for average muscle activity were taken from the middle 30-s sample of the 1 min for each condition	*Equipment:* surface electromyography device; *Variables:* RMS, that were expressed as a %sub-MVIC	*Muscle#1:* rectus abdominis; *Muscle#2:* transverse abdominis; *Muscle#3:* internal oblique; *Muscle#4:* iliocostalis lumborum pars thoracic; *Muscle#5:* lumbar multifidus *Electrode Location:* electrodes placed parallel to the muscle with a 2.5 cm center-to-center spacing	Transverse abdominis and internal oblique: significantly higher muscle activity compared to control at all time points, bilateral; Iliocostalis lumborum pars thoracic: the significant reduction compared to control during 12th-13th minute measurement; Internal oblique / rectus abdominis Ratio: significant improvement compared to control at all time points, bilateral; Lumbar multifidus/iliocostalis lumborum pars thoracic Ratio: higher ratios compared to control conditions bilaterally
Study: Holmes et al. [20]					
Sample Size: 16 participants (8 males and 8 females) Age Range: university-age Inclusion Criteria: No history of musculoskeletal injury or lower back pain in the past 12 months, as self-identified)	-Experimental Group: *Core Exercise Types*: forward–backward pelvic rotation; side-to-side pelvic rotation; circular hip rotation; alternating leg lifts; isometric side plank (as the reference exercise) *Experimental condition:* Core exercises on a stability ball Core exercises on a dynamic office chair Control Group: NA	Timepoint: during protocol Method: MVE were determined for each muscle using muscle-specific MVC. Each MVC involved a 3-s contraction, performed twice with a minimum 30-s rest between exertions Maximal contractions for the trunk extensors (TES, LES, MF) included a maximal back extension (modified Biering Sorensen test) For trunk flexors (RA, EO, IO) included Maximum forward-flexion contractions, right and left lateral bend, and right and left trunk twisting with flexion	Equipment: surface electromyograph device; *Variables:* Maximum and average muscle activities	-Three abdominal muscles bilaterally: *Muscle#1:* rectus abdominis; *Muscle#2:* external oblique; *Muscle#3:* internal oblique -three back muscles bilaterally: *Muscle#1:* thoracic erector spinae; *Electrode Location:* at the level of the T9 spinous process; *Muscle#2:* lumbar erector spinae; *Electrode Location:* at the level of the L1 spinous process; *Muscle#3:* multifidus; *Electrode Location:* at the level of the L4/ L5 spinous process) Pairs of electrodes were placed over each muscle belly, with a center-to-center interelectrode distance of 2 cm	Trunk muscle activity on the chair was similar to a stability ball for all exercises Higher peak activity in the right external oblique with the ball; Greater average activity in the left thoracic erector spinae on the chair compared to the ball
Study: Saiklang et al. [16]					
Sample Size: 30 participants (15 males and 15 females) Age Range: 20 to 39 years old Inclusion Criteria: ▪ CLBP lasting more than 3 months; ▪ Mild to moderate levels of pain; ▪ low levels of activity limitation; ▪ reporting sitting for at least 2 h on any working day	-Experimental Group: *Core Exercise Types:* dynamic lumbar extension with the abdominal drawing-in maneuver technique Participants engaged in the study for three consecutive days: Day 1: familiarization session; Day 2: group A completed the control condition, while Group B underwent the intervention condition; Washout period: 24 h; Day 3: participants switched roles -Control Condition: sitting without exercise;	Timepoint: during protocol Method: *trunk muscle activity* was measured simultaneously for 1 min at specific intervals (12–13, 25–26, and 38–39 min) *Trunk muscle fatigue data* were collected at intervals (0–10, 15–25, and 28–38 min) from the 41-min sitting period	Equipment: surface electromyography device; *Variables:* *For muscle fatigue*: MDF; *For muscle activity*: RMS	*Muscles:* 1. rectus abdominis; 2. internal oblique; 3. transversus abdominis; 4. iliocostalis lumborum pars thoracic; 5. lumbar multifidus; *Electrode Location:* eight pairs of electrodes, spaced 2.5 cm apart, were bilaterally attached to the above muscles	*For muscle fatigue*, the control condition demonstrated: significantly reduced MDF in transverse abdominis and internal oblique bilaterally at the second and third time of measurements (15th-25th and 28th-38th min) compared to the first measurement; Significantly reduced MDF in transverse abdominis and internal oblique bilaterally at the second and third time of measurements (15th-25th and 28th-38th min), and decreased MDF in lumbar multifidus bilaterally at the third time of measurement (28th-38th min) compared to the intervention condition *For muscle activity*, the intervention demonstrated significantly higher muscle activity than the control in transverse abdominis and internal oblique muscles bilaterally at each time

%sub-MVIC, Percentage of Sub-Maximal Voluntary Isometric Contraction; BMI, Body Mass Index; CLBP, Chronic Low Back Pain; MDF, Median Frequency; MVC, Maximal Voluntary Contraction; MVE, Maximal Voluntary Excitations; MVIC, Maximal Voluntary Isometric Contraction; NA, Not Applicable; RMS, Root Mean Square

*Muscle#* The numbered sequence of muscles examined in the study

**Table 4:** Summary of studies investigating effects of core exercise interventions on muscle activity

Participants	Intervention	Data collection	Equipment & variables	Measured muscles	Effect of core intervention on muscle activity
Study: Kim et al. [21]					
Sample Size: 29 females; Age Range: 20 to 23 years old; Inclusion Criteria: Chronic low back pain; Been physically inactive for more than six months	-Experimental Group: *Core Exercise Types:* 3D Moving Platform: Stretching and Strengthening Core & Paraspinal Muscles; *Duration:* 3 days/week for 8 weeks Control Group: without any intervention	Timepoint: pre-and post-intervention Method: Static Muscle Contraction: electric stimulation (10–65 mA, 1 ms) for isometric contractions; Dynamic Muscle Contraction: Range of Motion (-15° to 95°), 4 warm-up reps + 5 test reps at 30°/s for Pt, 4 warm-up reps + 15 test reps at 90°/s for Wr, 60s rest between tests	-Static Muscle Contraction: *Equipment:* tensiomyography device; *Variables:* Tc; Maximal Dm -Dynamic Muscle Contraction: *Equipment:* isokinetic device; *Variables:* Pt; Wr	-Static Muscle Contraction: *Muscle#1:* rectus abdominis; *Sensor & Electrode Location:* sensor tip at the navel, electrodes 3 cm apart on both sides; *Muscle#2:* erector spinae; *Sensor & Electrode Location:* sensor tip 5 cm above the lumbosacral joint, electrodes 3 cm apart on both sides -Dynamic Muscle Contraction: property in the abdomen and back	Static Muscle Contraction: increased right Tc of erector spinae in control compared to 3DEG, showing a significant time difference Dynamic Muscle Contraction: the 3D exercise group significantly increased trunk extensor work per repetition in trunk extensor
Study: Saiklan et al. [10]					
Sample Size: 30 participants (15 males and 15 females); Age Range*:* 20 to 39 years old Inclusion Criteria: ▪ CLBP lasting more than three months; ▪ mild to moderate levels of pain; ▪ low levels of activity limitation; ▪ reporting sitting for at least 2 h on any working day	-Experimental Group: *Core Exercise Types*: core stabilization exercise with the abdominal drawing-in maneuver technique *Duration:* 5 weeks, abdominal drawing-in maneuver, 41 min pre- and post-sitting -Control Group: NA	Timepoint: Pre- and post-intervention Method: muscle fatigue was assessed at intervals: 0–10, 15–25, and 28–38 min during 41 min of prolonged sitting	Equipment: surface electromyography device; *Variables:* MDF	*Muscles:* 1. rectus abdominis; 2. transverse abdominis; 3. internal oblique; 4. iliocostalis lumborum pars thoracis; 5. lumbar multifidus *Electrode Location:* electrodes were placed parallel to the muscle with a 2.5 cm center-to-center spacing	For both sides of the transverse abdominis and internal oblique muscles, the MDF value was significantly increased compared with the baseline values
Study: Yeom et al. [22]					
Sample Size: 50 sedentary adult male workers Age Range: 20 to 40 years old Inclusion Criteria: ▪ workers with a sedentary lifestyle, ▪ physical activity levels below the recommendations set by the WHO	-Experimental Group: *Core Exercise Types*: lumbar stabilization exercises *Duration:* 7 weeks, 3x/week, intensity increases -Control Group: NA	Timepoint: pre- and post-intervention Method: muscle measurements were conducted by applying electrical stimulation starting at 20 mA and increasing it by 20 mA until reaching the maximum contraction displacement of 100 mA. A 10-s rest period was allowed between each stimulus. Measurements were taken from right to left	Equipment: tensiomyography device *Variables:* Maximal Dm; the time taken to reach 10–90% of Dm (contraction time [Tc]); the time between the initiation of stimulus and 10% of Dm (delay time [Td]); the time of retention between 50% of Dm and 50% of the falling contour (sustain time [Ts]); the time taken to reach 90–50% of Dm from the falling contour (relaxation time [Tr])	*Muscle:* erector spinae muscle; *Sensor & Electrode Location:* sensor tip at the same level as the iliac crest, electrodes 2.5 cm apart on both sides	Tc and Td showed no significant difference, whereas Dm and Vc90 showed a significant increase after the completion of a 7-week core exercise program
Study: Lee et al. [23]					
Sample Size: 105 female office workers Age Range: aged ≥ 20 years., and average of 30.99 ± 10.85 years old Inclusion Criteria: ▪ No regular exercise performed in the past 6 months; ▪ Did not meet the WHO-recommended physical activity levels; ▪ Engaged in at least 7 h of sedentary work per day	-Experimental Group: *Core Exercise Types:* core stabilization exercises including Bracing and Hollowing Plank (side and prone), Hip Bridge, Back Extension, Bird dog, Trunk Twist *Duration:* 60 min/day, 3x/week for 7 weeks -Control Group: NA	Timepoint: pre- and post-intervention Method: Stimulus started at 20 mA and increased in 20 mA increments until maximum displacement. A 15 s rest period was observed between measurements, conducted from right to left	Equipment: tensiomyography device *Variables:* Tc; Maximum radial Dm; Mean velocity until 90% (Vc90)	*Muscle:* Erector spinae muscle; *Sensor & Electrode Location:* Sensor tip 5 cm above the PSIS, electrode pads 5 cm apart	There was a significant post-exercise increase in Dm and Vc90, but not Tc
Study: Jackson et al. [24]					
Sample Size: 12 participants (6 males and 6 females) Age Range: Males: 24.5 ± 3.7 years old; females: 23.2 ± 3.9 years old Inclusion Criteria: ▪ Participants who were regular computer users; ▪ Participants who were right-handed mousers; ▪ Participants who had not previously used a stability ball	-Experimental Group: *Core Exercise Types:* sitting on an Evolution Chair, which is a stability ball placed on a four-point base with casters *Duration:* 2 h/day, 9 days -Control Group: Did not use a stability ball during the time	Timepoint: Pre- and post-intervention Method: Muscle activation levels were measured in 14-min intervals throughout the 2-h sitting protocol, totaling eight blocks of data collection	Equipment: Surface electromyography device *Variables:* Mean muscle activation levels	*Muscle#1:* External oblique; *Electrode Location:* 15 cm lateral to the umbilicus; *Muscle#2:* Internal oblique; *Electrode Location:* below external oblique and superior to the inguinal ligament; *Muscle#3:* Thoracic erector spinae; *Electrode Location:* 5 cm lateral to T9 vertebrae; *Muscle#4:* Lumbar erector spinae; *Electrode Location:* 3 cm lateral to L3 vertebrae A reference electrode was placed over the right iliac crest. The electrodes were spaced apart by approximately 3 cm	No significant changes in muscle activation levels were observed between visits for both the accommodation and control groups across eight muscle sites There were no sex differences in average EMG amplitudes at any recording site Trunk muscle activity did not differ between groups

BMI, Body Mass Index; CLBP, Chronic Low Back Pain; Dm, Maximal Displacement; EMG, Electromyography; MDF, Median Frequency; MVC, Maximal Voluntary Contraction; MVE, Maximal Voluntary Excitations; NA, Not Applicable; Pt, Peak Torque; PSIS, Posterior Superior Iliac Spine; Tc, Contraction Time; WHO, World Health Organization; Wr, Work per Repetition

*Muscle#* The numbered sequence of muscles examined in the study

### Acute Effects of Core Exercise on Muscle Activity

The acute effects of core exercises on muscle activity were investigated in three studies [16, 19, 20]. All of them utilized an EMG system for evaluating muscle activity. Within this subset of studies, two studies measured root mean square [16, 19], and another reported both maximum and average muscle activity [20]. Two studies incorporated the abdominal drawing-in technique (also known in other studies as the hollowing technique) [16, 19], with one of them combining it with dynamic lumbar extension [16]. Additionally, one study evaluated the effectiveness of a dynamic office chair in activating trunk muscles during core exercises compared to a stability ball [20]. Table 3 summarizes the acute effects of core exercises on muscle activity.

### Effects of Core Exercise Interventions on Muscle Activity

Five out of nine studies examined the impact of core exercise interventions on muscle activity [10, 21–24]. Among these, three studies utilized tensiomyography [21–23] and reported parameters such as contraction time (Tc) [21–23] and maximal displacement (Dm) [21–23]. Two studies employed EMG [10, 24], one measuring median frequency [10], and the other reported the mean muscle activation level [24]. All five studies focused on core stability exercises [10, 21–24], with only one also assessing the impact of the abdominal drawing-in technique on muscle activity [10]. Table 4 offers an overview of studies investigating the effects of core exercise interventions on muscle activity.

## Discussion

Back pain is a common problem among sedentary employees who spend long periods sitting at work [44]. This prolonged sitting causes fatigue in the trunk muscles [10]. Under fatigue, muscular support to the spine decreases and increases stress on its passive structures [10]. This circumstance has become more prevalent due to the COVID-19 pandemic [8, 9]. To overcome this concern, one can employ recovery modalities in the workplace. Given the potential of core exercises to facilitate recovery from trunk muscle fatigue, this study summarized the evidence on the effects of core exercises in alleviating trunk muscular fatigue induced by prolonged sitting, focusing on objective outcomes such as electromyography and tensiomyography.

This scoping review examined eight studies: three focused on the muscle activity response to the core exercises [16, 19, 20], and five on the effects of core exercise interventions [10, 21–24].

During core exercises, fatigued trunk muscles exhibited an increase in EMG root mean square amplitude values. More specifically, two studies revealed that the abdominal drawing-in maneuver [16, 19] and a combination of the abdominal drawing-in maneuver and supported dynamic lumbar extension [16] enhanced the activation of the transversus abdominis and internal oblique muscles. The abdominal drawing-in technique also resulted in higher activation ratios of the transversus abdominis and internal oblique muscles compared to the rectus abdominis, indicating effective engagement of these crucial muscles for spinal support [19]. This maneuver reduced the activation of the lumbar multifidus muscle, particularly during its initial application [16]. This reduction may lead to decreased spinal compression forces, which is beneficial for individuals with chronic low back pain. Furthermore, in the intervention condition of the Saiklang et al. [16] study, no fatigue of the transversus abdominis and internal oblique muscles was observed throughout the 41-min testing period, while in the control condition, muscle fatigue occurred earlier, between the 15th and 25th minute of the sitting. This combination can improve muscle activity, and reduce muscle fatigue during prolonged sitting in people with chronic low back pain. However, these studies did not investigate the sex-specific tailoring of exercises to optimize outcomes. In a study conducted by Salim et al. [17], McKenzie’s exercises, including the Seated Lumbar Exercise and Standing Lumbar Extension Exercise, were found to reveal sex-specific differences in muscle activity. This was evident from EMG analysis of the erector spinae, latissimus dorsi, and internal and external oblique muscles. Following the Seated Lumbar Exercise, males showed reduced RMS values, whereas females exhibited increased values. After the Standing Lumbar Extension Exercise, males experienced a decline in RMS values across all muscles except the internal oblique, while females exhibited an increase in all muscles except the external oblique. This suggests that exercise interventions may need sex-specific tailoring to effectively prevent low back discomfort.

Furthermore, the choice of equipment, such as a stability ball or dynamic office chair, can influence muscle activation during core exercises. Across various core exercises, such as forward–backward pelvic rotation, side-to-side pelvic rotation, circular hip rotation, alternating leg lifts, and isometric side plank, muscle activity was comparable whether performed seated on a dynamic office chair or a stability ball [20]. The forward–backward pelvic rotation and circular hip rotation exercises generally showed the highest muscle activity levels. Notably, the stability ball induced greater peak activity in the right external oblique muscle, while the dynamic chair led to higher average activity in the left thoracic erector spinae. However, for most other muscles, activation levels were similar between the two conditions. This suggests that using a dynamic office chair in the workplace can help reduce the adverse effects of prolonged sitting, such as lower back pain.

In summary, diverse core exercises reveal specific patterns of muscle activation. The abdominal drawing-in maneuver enhances the activation of key trunk muscles such as the transversus abdominis, internal oblique, and erector spinae. These exercises may offer effective strategies for reducing muscle fatigue and preventing low back discomfort.

While the studies provide valuable insights into the acute effects of core exercises on muscle activity, future research should address several key areas. Firstly, there is a need to involve older office workers, as the current studies focused on young participants. Additionally, the current studies are mostly limited to those with low back pain, and it is crucial to include a group of healthy individuals to understand how exercises impact muscle response in those without a history of back pain. Moreover, the studies were conducted in laboratory settings, which may not fully reflect real workplace environments. Therefore, future research should explore the efficacy of interventions in actual settings. Furthermore, assessing the long-term sustainability of intervention benefits beyond their acute effects is essential for understanding their overall effectiveness (Table 5).

**Table 5:** Identified knowledge gaps in the muscle activity response to core exercises literature and recommendations for future studies

Identified knowledge gaps	Implications for future research
Inclusion of young sedentary individuals;	Future studies should involve older office workers to understand the effects of core exercises on muscle activity in this demographic
Predominant focus on individuals with low back pain;	It is crucial to include a group of healthy individuals in future research to examine how exercises impact muscle response in those without a history of back pain
Conducting studies in laboratory settings;	Future research should explore the efficacy of core exercise interventions in real workplace environments to ensure their practical applicability and relevance
Lack of assessment of long-term sustainability;	Evaluating the long-term sustainability of intervention benefits beyond acute effects is necessary to determine the overall effectiveness and durability of core exercises
Sex-specific tailoring of exercise interventions;	Future research should investigate the need for sex-specific tailoring of exercise interventions to effectively prevent low back discomfort and optimize outcomes

In the intervention studies, four reported significant improvements in outcome measures with targeted exercises [10, 21–23]. Specifically, an 8-week 3D moving platform program, incorporating stretching and strengthening exercises for core and paraspinal muscles, revealed increased activation of the paraspinal muscles, as evidenced by tensiomyography analysis [21]. Additionally, the intervention significantly improved static muscle contractions of the erector spinae and balanced the contraction times between the left and right sides of this muscle. A 5-week core stabilization exercise program targeted deep trunk muscles with the abdominal drawing-in maneuver technique during prolonged sitting [10]. Significant increases in MDF values were observed bilaterally for the transversus abdominis and internal oblique muscles after the 5-week intervention compared to baseline levels. This may be linked to the earlier onset of fatigue in these muscles during prolonged sitting. Both a 7-week lumbar stabilization exercise program [22] and a 7-week core stabilization exercise program [23] demonstrated significant increases in muscle stiffness (Dm) and contraction velocity of the erector spinae muscle. The increase in velocity of contraction at 90% (Vc90) suggested improved neuromuscular control, which is crucial for spine stability. The increase in Dm indicated enhanced muscle stiffness, potentially contributing to better spinal support. These findings were particularly relevant for sedentary workers who spend long hours sitting. However, a 9-day accommodation protocol resulted in no significant changes in targeted muscle activation levels, including the erector spinae, and internal, and external oblique muscles EMG [24]. In this protocol, participants engaged in two-hour sessions sitting on a stability ball with a four-point plastic base on casters.

Despite the valuable insights provided by these studies, there remains a notable gap in the literature. Research on long-term core exercise effects primarily targets the erector spinae muscle. However, it neglects the transversus abdominis and multifidus muscles, which are crucial for spinal stability during prolonged sitting [45]. While core exercises showed significant improvements in tensiomyography outcomes for the erector spinae, their effect on EMG remains uncertain, emphasizing the need for future evaluations. Similarly, studies examining the rectus abdominis using EMG and tensiomyography did not yield significant changes, indicating a need for further investigation. Conflicting outcomes have been observed regarding the effects of core exercise interventions on muscle parameters such as Dm, Vc90, and Tc for the erector spinae, highlighting the necessity for additional research. Two studies, one on core stabilization exercises and the other on lumbar stabilization exercises, demonstrated a significant increase in Dm and Vc90 for the erector spinae after core exercise interventions, but not in Tc. Conversely, a study involving a 3D Moving Platform with stretching and strengthening core and paraspinal muscles showed improvement in Tc but not in Dm and Vc90. This conflicting outcome emphasizes the necessity for further investigation. Additionally, there is insufficient attention given to assessing EMG mean frequency and root mean square values, which are essential for evaluating muscle fatigue and recovery. More emphasis should be placed on evaluating these values of electromyography signals to enhance the assessment of muscle fatigue and recovery (Table 6).

**Table 6:** Identified knowledge gaps in the literature related to the effect of core exercise interventions on muscle activity and recommendations for future studies

Identified knowledge gaps	Implications for future research
More focused on the erector spinae, neglecting transversus abdominis and multifidus;	Future studies should investigate the effects of core exercise interventions on the transversus abdominis and multifidus muscles, which are crucial for spinal stability during prolonged sitting;
Core exercise interventions show significant improvements in tensiomyography outcomes for the erector spinae, uncertain for electromyography;	Further research is needed to clarify the impact of core exercise interventions on electromyography values, providing a comprehensive understanding of their effects on muscle activity
No significant changes in rectus abdominis with electromyography and tensiomyography	There is a need for additional investigation into the effects of core exercise interventions on the rectus abdominis muscle, utilizing both electromyography and tensiomyography methods to obtain a more comprehensive understanding of muscle activity changes
Conflicting outcomes are observed regarding the effects of core exercise interventions on muscle parameters such as Dm, Vc90, and Tc for the erector spinae	Future studies should aim to reconcile conflicting outcomes and provide clarity on the effects of core exercise interventions on muscle parameters, such as Dm, Vc90, and Tc, for the erector spinae muscle
Insufficient attention is given to assessing the mean frequency and root mean square values of electromyography signals	There is a need for future research to incorporate assessments of mean frequency and root mean square values of electromyography signals, which are essential for evaluating muscle fatigue and recovery

Dm, Maximal Displacement; Vc90, Velocity of Contraction at 90%; Tc, Contraction Time

## Conclusion

Core exercises are effective in alleviating muscular fatigue caused by prolonged sitting. This can be observed from significant changes in EMG and tensiomyography parameters during exercise and after the training protocol. Future research should include older office workers, and real workplace settings to understand the efficacy of exercises in practical environments. While current studies predominantly focus on the recovery of the erector spinae muscles, overlooking other trunk muscles like the transversus abdominis and multifidus indicates a critical gap in research. Despite observed improvements in tensiomyography outcomes of erector spinae muscles, uncertainties persist regarding EMG post-core exercise interventions. The neglect of the transversus abdominis and multifidus muscles in long-term core exercise research further underscores the need for more inclusive investigation. Furthermore, conflicting findings regarding muscle parameters highlight the necessity for additional studies. Addressing these gaps would provide a clearer understanding of effective core exercise intervention for mitigating muscular fatigue in sedentary individuals engaged in prolonged sitting.

## Data Availability

Not applicable.

## Abbreviations

%sub-MVIC: Percentage of Sub-Maximal Voluntary Isometric Contraction
BMI: Body Mass Index
CLBP: Chronic Low Back Pain
Dm: Maximal Displacement
EMG: Electromyography
MDF: Median Frequency
MVC: Maximal Voluntary Contraction
MVE: Maximal Voluntary Excitations
MVIC: Maximal Voluntary Isometric Contraction
NA: Not Applicable
Pt: Peak Torque
PSIS: Posterior Superior Iliac Spine
RA: Rectus Abdominis
RMS: Root Mean Square
Tc: Contraction Time
WHO: World Health Organization
Wr: Work per Repetition
